# *DOK6* promoter methylation serves as a potential biomarker affecting prognosis in de novo acute myeloid leukemia

**DOI:** 10.1002/cam4.2540

**Published:** 2019-09-04

**Authors:** Guo‐Kang Sun, Li‐Juan Tang, Jing‐Dong Zhou, Zi‐Jun Xu, Lan Yang, Qian Yuan, Ji‐Chun Ma, Xing‐Hui Liu, Jiang Lin, Jun Qian, Dong‐Ming Yao

**Affiliations:** ^1^ Laboratory Center Affiliated People's Hospital of Jiangsu University Zhenjiang Jiangsu People's Republic of China; ^2^ The Key Lab of Precision Diagnosis and Treatment of Zhenjiang City Zhenjiang Jiangsu People's Republic of China; ^3^ The Key Lab of Precision Diagnosis and Treatment in Hematologic Malignancies of Zhenjiang City Zhenjiang Jiangsu People's Republic of China; ^4^ Department of Clinical Laboratory Shanghai Gongli Hospital The Second Military Medical University Shanghai China; ^5^ Department of Hematology Affiliated People's Hospital of Jiangsu University Zhenjiang Jiangsu People's Republic of China

**Keywords:** AML, biomarker, *DOK6*, methylation, prognosis

## Abstract

**Background:**

Downstream of tyrosine kinase 6 (*DOK6*), which is specifically expressed in the nervous system, was previously recognized as an adapter only in neurite outgrowth. Recent studies also demonstrated the potential role of *DOK6* in solid tumors such as gastric cancer and breast cancer. However, previous studies of *DOK6* have not dealt with its roles in myeloid malignancies. Herein, we verified the promoter methylation status of *DOK6* and further explored its clinical implication in de novo acute myeloid leukemia (AML).

**Methods:**

A total of 100 newly diagnosed adult AML patients were involved in the current study. *DOK6* expression and methylation were detected by real‐time qPCR and methylation‐specific PCR (MSP), respectively. Bisulfite sequencing PCR (BSP) was performed to assess the methylation density of the *DOK6* promoter.

**Results:**

Downstream of tyrosine kinase 6 promoter methylation was significantly increased in AML patients compared to controls (*P* = .037), whereas *DOK6* expression significantly decreased in AML patients (*P* < .001). The expression of *DOK6* was markedly up‐regulated after treated by 5‐aza‐2′‐deoxycytidine (5‐aza‐dC) in THP‐1 cell lines. The methylation status of the *DOK6* promoter was associated with French‐American‐British classifications (*P* = .037). There was no significant correlation existed between *DOK6* expression and its promoter methylation (*R* = .077, *P* = .635). Interestingly, of whole‐AML and non‐APL AML patients, both have a tendency pertaining to the *DOK6* methylation group and a significantly longer overall survival (OS) than the *DOK6* unmethylation group (*P* = .042 and .036, respectively).

**Conclusion:**

Our study suggested that *DOK6* promoter hypermethylation was a common molecular event in de novo AML patients. Remarkably, *DOK6* promoter methylation could serve as an independent and integrated prognostic biomarker not only in non‐APL AML patients but also in AML patients who are less than 60 years old.

## Graphical abstract

DOK6; Methylation; AML.

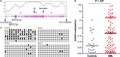

## 1 BACKGROUND

As a disease characterized by clonal hematopoietic stem cell disorders, acute myeloid leukemia (AML) has a cure rate of 35%‐40% in those younger than 60 and a cure rate of 5%‐15% in those over 60 years of age.^1^ However, there were only 5‐10 months of median survival in older patients who could not tolerate the side effects of intensive chemotherapy.^2^ Despite the molecular diagnosis and chemotherapy improvements, the long‐term survival rate for patients with advanced stage remains disappointing.^3^ Currently, the molecular evaluation that focused on a single consistent cancer pathway for intensive induction chemotherapy or complete remission in AML seems to be weak. Additionally, the cancer phenotype typically is kept by multiple oncogenic pathways or processes.^4^ Thus, newly integrated biomarkers which act as modulators for multiple oncogenic signaling pathways are urgently needed.

Downstream of tyrosine kinase (*DOK*) multigenic family consists of seven family members, which possess a similar structural topology and function as substrates of nonreceptor tyrosine kinases and multiple receptor tyrosine kinases.^5^, ^6^, ^7^, ^8^ Some of them have been proved to play a key role in the negative regulation of immune cell signaling.^6^, ^9^, ^10^, ^11^ For example, *DOK*1, *DOK*2, and *DOK*3 were identified as a tumor suppressor in lung tumor and aggressive histiocytic sarcoma (HS).^12^, ^13^, ^14^ Downstream of tyrosine kinase 4 and *DOK*5 is mainly expressed in the nervous system.^15^, ^16^ However, *DOK7* was mainly enriched in skeletal muscle and myocardium.^17^ Previously, *DOK6* was found to be involved in neuronal development through Ret and neurotrophin‐3 signaling.^18^, ^19^, ^20^ Leong et al showed that *DOK6* is involved in a variety of oncogenic signaling pathways and functioned broadly in gastric cancer, and provided functional relevance of its binding to the epidermal growth factor receptor (EGFR).^21^ Tamara et al reported that *DOK6* behaved as a tumor suppressor in human breast cancer.^22^ However, the research to date has tended to focus on solid tumors rather than the hematological tumor. The expression of *DOK6* remains unknown. Furthermore, whether *DOK6* expression is regulated by its promoter region in which a large CpG island is embedded is still unknown. This prompted us to investigate the methylation status of the *DOK6* promoter and further explore its clinical significance in AML patients.

## 2 MATERIALS AND METHODS

### 2.1 Cell cultures

In this study, the leukemia cell line THP‐1 was cultured using RPMI 1640 medium with a serum concentration of 10% fetal calf. An environment having a temperature of 37°C and a carbon dioxide concentration of 5% was set as the cell culture condition. For demethylation experiments, cells were treated by a final concentration of 0, 0.1, 1 and 10 μmol/L 5‐aza‐dC (Sigma Aldrich) for 72 hours before harvest.

### 2.2 Patients and tissue samples

Bone marrow (BM) specimens from 100 patients were collected for genomic DNA extraction. All patients had a confirmed diagnosis of previously untreated AML at the Affiliated People's Hospital of Jiangsu University, Jiangsu, China. Normal BM samples were picked up from 23 healthy donors. The diagnosis and clinical stages of AML were confirmed following the French‐American‐British (FAB) and the World Health Organization (WHO) criteria.^23^, ^24^ All eligibility criteria and treatment protocols were consistent with our previous reports.^25^ Lymphocyte Separation Medium and gradient centrifugation were used to extract BM mononuclear cells (BMMNCs) from BM specimens. The study was approved by the Clinical Research Ethics Committee of the Affiliated People's Hospital of Jiangsu University and all patients signed informed consent for voluntary participation.

### 2.3 RNA isolation, reverse transcription, and real‐time qPCR

Trizol reagent (Invitrogen) was used to isolate total RNA from pre‐extracted BMMNCs. Reverse transcription reaction with 40 μL volume was composed of 10 mmol/L of dNTPs (deoxyribonucleoside triphosphates), 5× buffer 10 mmol/L, 80 U of RNAsin, 10 μmol/L of random hexamers, and 200 U of MMLV reverse transcriptase (Eppendorf). The reaction conditions were incubated for 10 minutes at 25°C, 60 minutes at 42°C, and then stored at −20°C. Analysis of *DOK6* gene expression in AML and control specimens was performed by real‐time qPCR with the primers shown in Table 1. The real‐time qPCR reaction system with 20 μL volume composed of cDNA 20 ng, 0.8 μmol/L of primers, 0.4 μmol/L of ROX Reference Dye II (Takara), and 10 μmol/L of SYBR Premix TB Green. The real‐time qPCR reaction conditions were 95°C for 5 minutes, followed by 40 cycles at 95°C for 10 seconds, 60°C for 30 seconds, 72°C for 30 seconds, and 82°C for 30 seconds to collect fluorescence, finally followed by 95°C for 15 seconds, 60°C for 60 seconds, 95°C for 15 seconds, and 60°C for 15 seconds. Negative and positive controls were included to rule out false positives and false negatives, respectively. The relative expression levels of *DOK6* were calculated by the 2^−ΔΔCT^ method.

**Table 1:** Primers used for qPCR, MSP and BSP

Primers		Sequence(5′‐3′)	Product size (bp)
qPCR	*DOK6*‐Forward	CAGGGCTACGTGAAAATCCG	200
	*DOK6*‐Reverse	TTCTTTGTCTCTCGGGGCAG	
MSP	*DOK6*‐M‐Forward	ATTAATTATTCGGGTCGGTC	128
	*DOK6*‐M‐Reverse	AAAAAAACCAATCGTACGC	
	*DOK6*‐U‐Forward	TAAATTAATTATTTGGGTTGGTT	128
	*DOK6*‐U‐Reverse	CACAAAAAAACCAATCATACAC	
BSP	*DOK6*‐B‐Forward	TTATGTGTTTTTATATTAAGGGGAGAA	312
	*DOK6*‐B‐Reverse	CAAACCCTTCCTAATACACACA	

Abbreviations: BSP, bisulfite sequencing PCR; *DOK6*, downstream of tyrosine kinase 6; M, methylation; MSP, real‐time quantitative methylation‐specific PCR; qPCR, real‐time quantitative PCR; U, unmethylation.

### 2.4 DNA extraction, bisulfite modification and methylation‐specific PCR

Genomic DNA from AML patients, AML cultured cells and healthy donors were isolated using genomic DNA purification kit (Gentra). The CpGenome DNA Modification Kit (Chemicon) was used to modify genomic DNA according to the manufacturer's recommendations. Methylation‐specific PCR (MSP) was used to detect *DOK6* methylation status by the methylation primers (Table 1) with SYBR Premix Ex TaqII (Takara). The reaction conditions were 95°C for 30 seconds, 40 cycles for 5 seconds at 95°C, 30 seconds at 62°C, 30 seconds at 72°C, and 78°C for 32 seconds. DNA bisulfite modification was carried out using the CpGenome™ DNA Modification Kit (Chemicon). The quantification of *DOK6* methylation was calculated with the same model as *DOK6* expression.

### 2.5 Bisulfite sequencing PCR

For bisulfite sequencing PCR (BSP), a 312‐bp fragment was amplified from the *DOK6* promoter region, using primers pair specific for bisulfite‐modified sequences (Table 1). Bisulfite sequencing PCR reaction conditions were 98°C for 10 seconds, 40 cycles for 10 seconds at 98°C, 30 seconds at 59°C, 72°C for 30 seconds, and followed by a final 7 minutes extension step at 72°C. The reaction system of BSP was carried out as reported previously.^26^, ^27^ AxyPrep DNA gel extraction kit (AxyGen) was used to purify BSP products, ligated into pMD 19‐T Vector (Takara), and then transfected into DH5α competent cells (Vazyme) for cloning. Finally, six independent clones of each sample were sequenced timely (BGI Tech Solutions Co.).

### 2.6 Statistical analysis

All data were analyzed using IBM SPSS software package version 22.0 and GraphPad Prism 5.0. The Pearson Chi‐square test or Fisher exact test was applied to compare two groups of categorical variables. Student's *t* test was applied to compare two groups for normally distributed quantitative variables. Kaplan‐Meier analysis and Cox regression model (univariate and multivariate analyses) were used to assess the effect of *DOK6* methylation on the overall survival (OS). A two‐sided *P* value of .05 or less was defined as statistically significant.

## 3 RESULTS

### 3.1 The methylation of *DOK6* promoter in AML patients at diagnosis

**Figure 1:**
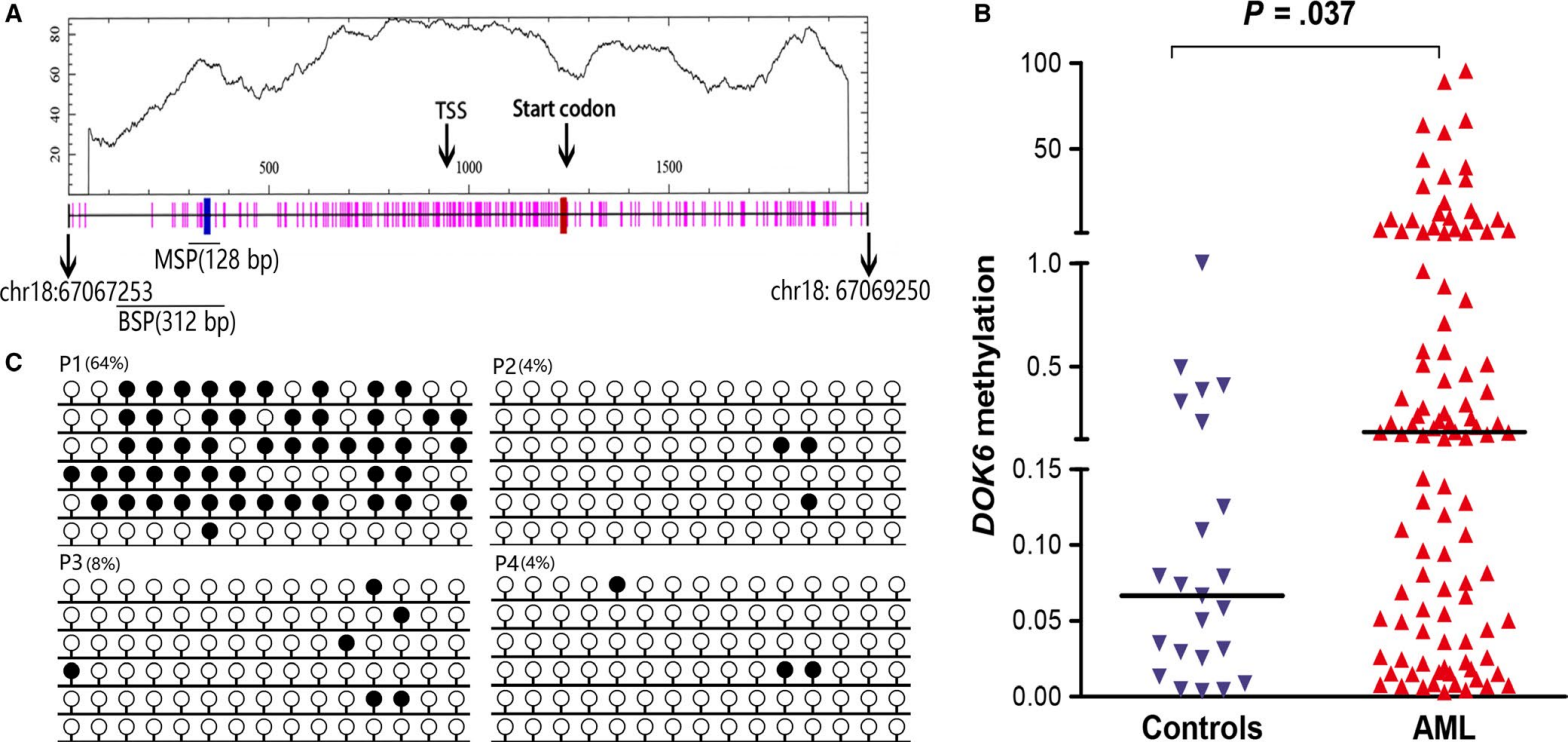
Downstream of tyrosine kinase 6 (*DOK6*) methylation primer position and methylation density of the *DOK6* promoter in AML. A, The genomic coordinates (GC) of *DOK6* promoter region CpG island and primer locations. The panel plots the GC content as a percentage of the total. Each vertical bar in the bottom panel represents the presence of a CpG dinucleotide. Black horizontal bars indicate regions amplified by MSP primer pairs and BSP primer pairs. This figure was created using Methyl Primer Express v1.0 software and CpGplot (http://emboss.bioinformatics.nl/cgi-bin/emboss/cpgplot). AML, acute myeloid leukemia; BSP, bisulfite sequencing PCR; MSP, methylation‐specific PCR; TSS, transcription start site. B, Relative promoter methylation level of *DOK6* in AML patients and controls. *DOK6* methylation level was examined by MSP *DOK6* methylation level was up‐regulated in AML patients compare to controls. MSP: methylation‐specific PCR. C, Methylation density of *DOK6* promoter in AML patients and controls. Methylation density was determined by BSP. White cycle: unmethylated CpG dinucleotide; Black cycle: methylated CpG dinucleotide. P1: methylated AML patient; P2: unmethylated AML patient; P3 and P4: controls

To examine the promoter methylation status of *DOK*6 in AML patients and further analyze their clinical significance, the MSP and BSP primer sets and assays were designed at the CpG islands of the *DOK6* gene promoter (Figure 1A). Firstly, *DOK6* methylation status was examined by MSP, and the results showed that the *DOK6* promoter methylation level of AML patients is significantly higher than controls, with a median of 0.231 vs 0.060 (*P* = .037; Figure 1B). Secondly, two controls and a *DOK6* methylated AML patient, as well as a *DOK6* unmethylated AML patient, were selected randomly to verify the MSP results by BSP. Consistent with the result of MSP, both the *DOK6* promoter of healthy donors and the unmethylated patient tend to present completely unmethylated, while the methylated AML patient demonstrated a high methylation density (Figure 1C). In addition, *DOK6* promoter methylation was significantly decreased in MDS and CML patients compared to controls (*P* = .0002 and *P* < .0001, respectively; Additional file 1: Figure S1).

### 3.2 Epigenetic mechanism regulating *DOK6* expression in AML

**Figure 2:**
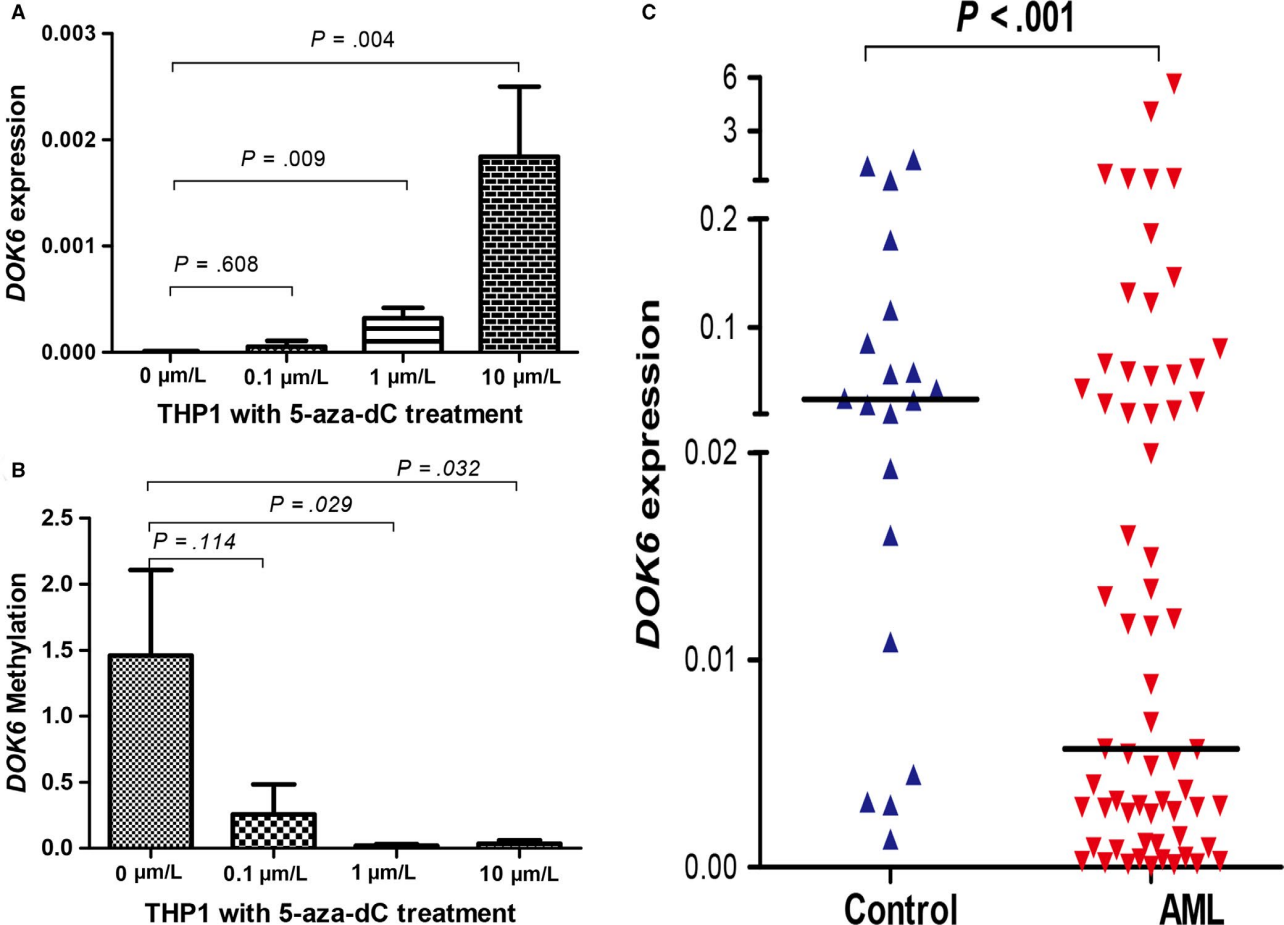
Downstream of tyrosine kinase 6 (*DOK6*) expression levels in THP‐1 cell line and acute myeloid leukemia (AML). A, *DOK6* relative expression in THP‐1 cell line with or without 5‐aza‐dC treatment; *DOK6* expression level was examined by real‐time quantitative PCR (qPCR). B, *DOK6* promoter methylation levels in THP‐1 cell line with or without 5‐aza‐dC treatment; *DOK6* methylation level was examined by methylation‐specific PCR. C, Relative expression level of *DOK6* in AML patients and controls. *DOK6* expression level was examined by qPCR

To identify whether *DOK6* expression is regulated by its promoter methylation in AML, 5‐aza‐dC, the DNMT inhibitor, was used to treat the THP‐1 cell line. The expression of *DOK6* was markedly up‐regulated after 5‐aza‐dC treatment (Figure 2A). Meanwhile, the methylation level of the *DOK6* promoter was significantly decreased in THP‐1 cell lines which were treated by 5‐aza‐dC (Figure 2B). Additionally, a small quantity of AML samples was used to detect the expression of *DOK6* in the current study. The results showed that *DOK6* significantly decreased in de novo AML patients (*P* < .001; Figure 2C).

### 3.3 Comparison of clinical characteristics between *DOK6* unmethylated and *DOK6* methylated group

To further analyze the clinical impact of *DOK6* methylation, all patients of AML were divided into *DOK6* unmethylated and *DOK6* methylated groups according to the cutoff value. No significant differences were observed in variables including sex, age, white blood cell, platelets, hemoglobin, and BM blasts between the patients with and without *DOK6* promoter methylation (*P* > .05; Table 2). Moreover, there was no significant difference in karyotypic classifications between the methylated and unmethylated patients (*P* > .05; Table 2). However, the gene mutation of nucleophosmin (NPM1) and isocitrate dehydrogenase (IDH1/2) was more frequently observed in unmethylated patients (*P* = .075, and .075, respectively; Table 2). Moreover, statistical analysis showed a significant difference in the distribution of FAB between the methylated and unmethylated patients (*P* = .037; Table 2).

**Table 2:** Comparison of clinical characteristics between *DOK6* unmethylated and *DOK6* methylated group

Patient's parameters	Methylated (n = 52)	Unmethylated (n = 48)	*P* value
Sex, male/female	31/21	28/20	>.999
Median age, y (range)	49.00 (18‐80)	56.5 (18‐85)	.320
Median WBC, ×10^9^/L (range)	9.250 (0.3‐528.0)	19.00 (0.4‐129.1)	.988
Median platelets, ×10^9^/L (range)	37.50 (5‐264)	40 (9‐191)	.753
Median hemoglobin, g/L (range)	72.5 (34‐123)	82.5 (32‐135)	.417
BM blasts, % (range)	40.0 (1.0‐97.5)	43.0 (6.5‐99.0)	.529
FAB			.037
M0	0	2	
M1	5	0	
M2	20	18	
M3	16	8	
M4	6	13	
M5	3	5	
M6	2	2	
Karyotype classification			.162
Favorable	18 (35%)	12 (25%)	
Intermediate	24(46%)	24 (50%)	
Poor	10 (19%)	8 (16%)	
No data	0 (0%)	4 (8%)	
Karyotype			.379
1Normal	20 (38%)	18(38%)	
2t(8;21)	4 (8%)	4 (8%)	
3t(15;17)	14 (27%)	8 (17%)	
4t(9;22)	1 (2%)	1 (2%)	
+8	0 (0%)	2 (4%)	
−7/7q−	1 (2%)	0(0%)	
5complex	4 (8%)	4 (8%)	
6others	8 (15%)	7 (15%)	
7No data	0 (0%)	4 (8%)	
Gene mutation			
*CEBPA* (+/−)	4/40	2/31	.695
*NPM1* (+/−)	0/44	3/30	.075
*FLT3*‐ITD (+/−)	3/41	1/32	.631
*C‐KIT*(+/−)	3/41	1/32	.631
*N/K‐RAS* (+/−)	2/42	0/33	.504
*IDH1/2* (+/−)	0/44	3/30	.075
*DNMT3A* (+/−)	2/42	1/32	>.999
*U2AF1* (+/−)	2/42	0/33	.504
CR (−/+)	23/25	26/16	.208

Abbreviations: BM, bone marrow; CR, complete remission; *DOK6*, downstream of tyrosine kinase 6; FAB, French‐American‐British; WBC, white blood cells.

### 3.4 Prognostic significance of *DOK6* promoter methylation in whole‐AML and non‐APL patients

**Figure 3:**
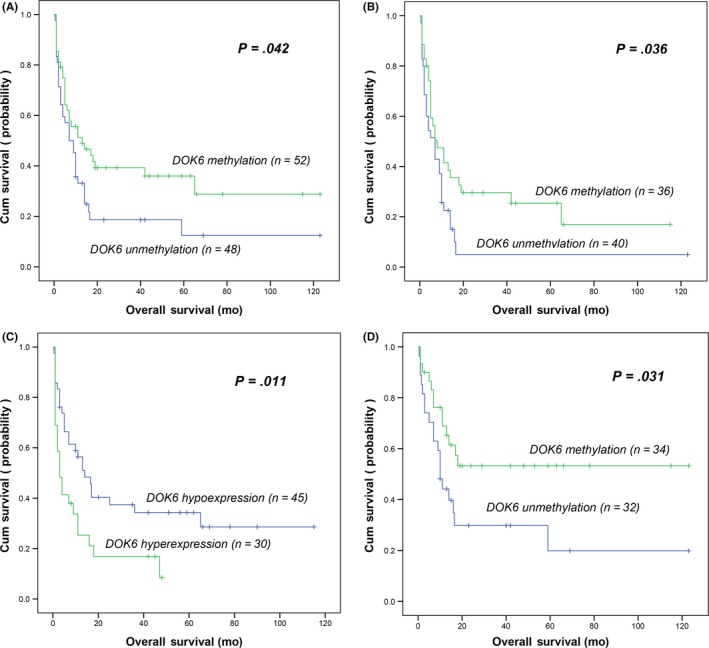
Prognostic value of downstream of tyrosine kinase 6 (*DOK6*) methylation in acute myeloid leukemia (AML) patients. (A, B, D) For *DOK6* methylation in AML patients and non‐APL AML patients as well as AML patients who are less than 60 years old. C, For *DOK6* expression in de novo AML patients. *DOK6* expression level was examined by real‐time quantitative PCR

To determine the prognostic value of *DOK6* promoter methylation in AML, a total of 100 cases with follow‐up data were used for survival analysis. No significant differences were observed in the complete remission (CR) rate between patients with and without *DOK6* promoter methylation (52% vs 38%; *P* = .208). However, in whole‐AML cases, patients with *DOK6* promoter methylated had a significantly longer OS than those without *DOK6* promoter methylated (mean 23.10 vs 14.20 months; *P* = .042; Figure 3A). Furthermore, among non‐APL patients, the patients with *DOK6* promoter methylation also had significantly longer OS than those without *DOK6* promoter methylation (mean 19.17 vs 9.96 months; *P* = .036; Figure 3B). To check out the independent prognostic factors on disease outcome in non‐APL AML, a multivariate logistic analysis model was created (Table 3). Downstream of tyrosine kinase 6 promoter methylation was one of the independent factors which displayed an approximatively significant impact on OS (odds ratio [OR] = 0.577, 95% confidence interval [CI] [0.331‐1.005], *P* = .052) in non‐APL patients, other factors associated with OS were age and karyotype risk (Table 3). In addition, *DOK6* low‐expression patients had a significantly longer OS (*P* = .011; Figure 3C).

**Table 3:** Univariate and multivariate analysis of prognostic factors for overall survival in non‐APL patients

Variables	Overall survival			
	Univariate analysis		Multivariate analysis	
	HR (95% CI)	*P* value	HR (95% CI)	*P* value
WBC	1.852 (1.098‐3.123)	.021	1.506 (0.883‐2.568)	.133
Age	2.139 (1.262‐3.625)	.005	2.081 (1.213‐3.570)	.008
*DOK6 methylation*	0.580 (0.339‐0.993)	.047	0.577 (0.331‐1.005)	.052
Karyotype risk	1.834 (1.252‐2.686)	.002	1.618 (1.107‐2.365)	.013
*FLT3*‐ITD mutation	0.830 (0.255‐2.701)	.757	—	—
*NPM1* mutation	0.660 (0.158‐2.751)	.568	—	—
*CEBPA* mutation	0.746 (0.180‐3.094)	.686	—	—
*c‐KIT* mutation	0.309 (0.042‐2.246)	.246	—	—
*N/K‐RAS* mutation	0.421 (0.057‐3.100)	.396	—	—
*IDH1/2* mutation	0.960 (0.227‐4.052)	.956	—	—
*DNMT3A* mutation	1.183 (0.363‐3.856)	.780	—	—

Note

Variables including age (≤60 vs >60 years), WBC (≥30 × 10^9^ vs <30 × 10^9^/L), *DOK6 methylation* (unmethylated vs methylated), karyotype risk (favorable vs intermediate vs poor), and gene mutations (mutant vs wild‐type).

Multivariate analysis includes variables with *P* < .200 in univariate analysis.

Abbreviations: CI, confidence interval; *DOK6*, downstream of tyrosine kinase 6; HR, hazard ratio.

### 3.5 Prognostic significance of *DOK6* promoter methylated in AML patients who are less than or equal 60 years old

Because age is usually treated as an important risk factor in cancer, patients who are less than 60 years old were separated in the this study. Similarly, significant difference also was found in OS between the patients with and without *DOK6* promoter methylation (mean 29.77 vs 19.31 months; *P* = .031; Figure 3D). Multivariate Cox analysis identified *DOK6* methylation as an independent prognostic factor (OR = 0.477, 95% CI [0.233‐0.976] *P* = .043) (Table 4).

**Table 4:** Univariate and multivariate analysis of prognostic factors for overall survival in AML patients who are less than or equal 60 years old

Variables	Overall survival			
	Univariate analysis		Multivariate analysis	
	HR (95% CI)	*P* value	HR (95% CI)	*P* value
WBC	2.185 (1.052‐4.540)	.036	1.302 (0.593‐2.859)	.511
*DOK6* methylation	0.473 (0.232‐0.961)	.038	0.477 (0.233‐0.976)	.043
Karyotype risk	2.840 (1.781‐4.527)	<.001	2.840 (1.769‐4.561)	<.001
*NPM1* mutation	1.236 (0.292‐5.237)	.774	—	—
*CEBPA* mutation	1.172 (0.277‐4.968)	.829	—	—
*c‐KIT* mutation	0.710 (0.096‐5.257)	.738	—	—
*N/K‐RAS* mutation	0.835 (0.113‐6.173)	.860	—	—
*IDH1/2* mutation	0.996 (0.135‐7.381)	.997	—	—
*DNMT3A* mutation	1.101 (0.148‐8.166)	.925	—	—

Note

Variables including WBC (≥30 × 10^9^ vs <30 × 10^9^/L), *DOK6 methylation* (unmethylated vs methylated), karyotype risk (favorable vs intermediate vs poor), and gene mutations (mutant vs wild‐type).

Multivariate analysis includes variables with *P* < .200 in univariate analysis.

Abbreviations: CI, confidence interval; *DOK6*, downstream of tyrosine kinase 6; HR, hazard ratio.

## 4 DISCUSSION

Downstream of tyrosine kinase family, which acts as substrates of multiple receptor tyrosine kinases and nonreceptor tyrosine kinases, plays a unique role in different organs and tissues.^28^ All family members display a high degree of similarity over the regions, in which the Pleckstrin homology and phosphotyrosine‐binding (PTB) domains existed.^29^ Interestingly, despite the fact that all members of the *DOK* family share similar structure, they exert differently, or even opposite, roles based on the surrounding circumstances.^15^, ^17^, ^30^, ^31^ As a sort of adapter with multiple docking sites for signaling proteins, *DOK* proteins act as both carcinogenic and tumor‐suppressing proteins. Recently, He et al have proved that the expression of *DOK1/2* was inactivated by their promoter methylation, and is associated with an adverse prognosis in AML.^32^ The ' study by Fu et al has shown that increased *DOK4* and *DOK5* expression were closely related to adverse prognosis, while increased *DOK7* expression was associated with a favorable prognosis in AML.^33^ The above literature data demonstrated that different *DOK* protein exerts a different effect on OS and LFS in AML.

Downstream of tyrosine kinase 6, among them, was found to promote neurite outgrowth by the Ret‐mediated signaling pathway in N2A‐α1 cells.^18^ Wei et al demonstrated that *DOK6* selectively combined with the NPQY motif of TrkC via its PTB domain in a kinase activity‐dependent manner and is involved in NT‐3‐mediated neuronal development.^20^ Besides, Leong and his colleagues reported that *DOK6* combined with various components in different steps of multiple signaling pathways, such as platelet‐derived growth factor, nerve growth factor, EGFR, RAS, vascular endothelial growth factor and RAF/MAP kinase.^21^ Importantly, most of them had been proved as carcinogenic proteins and adverse prognostic factors in gastric cancer.^34^, ^35^, ^36^, ^37^ Accumulating results imply that *DOK6* enhances many oncogenic signaling pathways by interacting with a variety of different signaling proteins and receptors. Therefore, with the reduction of *DOK6* expression, multiple carcinogenic signaling pathways would be inevitably affected.

As the most studied epigenetic alteration, DNA methylation has been involved in a variety of regulatory processes, such as genome integrity, loss of imprinting, genome integrity, transcriptional regulation, and chromatin structure.^38^ Therefore, cancer‐specific promoter methylation contributes to the discovery of novel tumor suppressor genes and/or tumor‐specific prognostic biomarkers, the development of novel treatment strategies, and treatment response prediction. Here, as far as we know, it is the first time to report that *DOK6* promoter methylation was a common event in patients with newly diagnosed AML. Although we did not observe the significant impact of *DOK6* promoter methylation on CR, our investigation revealed that the methylation status of the *DOK6* promoter had a significant association with OS. Interestingly, patients with *DOK6* promoter methylation displayed a much longer OS in both whole‐AML and non‐APL patients. Notably, our results referring to the prognostic value of *DOK6* expression were consistent with those reported in gastric cancer by Leong et al^21^ Similar prognostic value of the other *DOK* family member such as *DOK4/5* was also reported by Fu et al.^17^ A possible explanation for this was that decreased *DOK6* expression affected multiple carcinogenic signaling pathways, which contributed to the favorable outcome of methylated AML patients. Further research should be taken to expand the molecular mechanisms involved in *DOK6* adaptor protein's function in multiple tyrosine kinases signaling pathways as well as their role in leukemogenesis.

As is well known, DNA methylation in promoter CpG islands played a crucial role in regulating gene expression. In this study, we also revealed that *DOK6* was significantly decreased in de novo AML patients and decreased *DOK6* expression was associated with a favorable outcome. Furthermore, the cell experiment indicated that 5‐aza‐dC increased *DOK6* expression in leukemia cells THP‐1 by inducing demethylation of the *DOK6* promoter region.

## 5 CONCLUSION

Taken together, our study identified that *DOK6* promoter methylation is a common molecular event in de novo AML patients. Remarkably, *DOK6* promoter methylation could serve as an independent and integrated prognostic biomarker not only in non‐APL but also in AML patients who are less than or equal 60 years old.

## DECLARATIONS

### Ethics approval and consent to participate

The study was approved by the Clinical Research Ethics Committee of the Affiliated People's Hospital of Jiangsu University.

### Consent for publication

Written informed consents were obtained from all enrolled voluntary individuals before their participation.

## ACKNOWLEDGMENTS

The authors sincerely thank all the patients and their families for participating in this project.

## CONFLICT OF INTEREST

None declared.

## Data Availability

The datasets used and/or analyzed during the current study are available from the corresponding author on reasonable request.
