# Supplementary material for: DOK6 promoter methylation serves as a potential biomarker affecting prognosis in de novo acute myeloid leukemia
Source: Cancer Med. 2019 Sep 4;8(14):6393–402. doi: 10.1002/cam4.2540 (PMC6797566; doi:10.1002/cam4.2540)
Supplement: Supplementary file 1 [file CAM4-8-6393-s001.docx]

**Figure S1. The relative promoter methylation level of *DOK6* in MDS and CML patients compared to controls. *DOK6* methylation level was examined by MSP. (a):** *DOK6* methylation levels were lower in MDS patients compared to controls. **(b):** *DOK6* methylation levels were lower in CML patients compared to controls.
